# 多接收器电感耦合等离子体质谱方法的开发和应用进展

**DOI:** 10.3724/SP.J.1123.2020.07030

**Published:** 2021-01-08

**Authors:** Luyao ZHANG, Zigu CHEN, Xuezhi YANG, Dawei LU, Qian LIU, Guibin JIANG

**Affiliations:** 1.中国科学院生态环境研究中心, 环境化学与生态毒理学国家重点实验室, 北京 100085; 1. State Key Laboratory of Environmental Chemistry and Ecotoxicology, Research Center for Eco-Environmental Sciences, Chinese Academy of Sciences, Beijing 100085, China; 2.中国科学院大学资源与环境学院, 北京 100190; 2. College of Resources and Environment, University of Chinese Academy of Sciences, Beijing 100190, China

**Keywords:** 多接收器电感耦合等离子体质谱, 离子交换色谱, 同位素分析, 样品净化, 联用技术, multi-collector inductively coupled plasma mass spectrometry (MC-ICP-MS), ion-exchange chromatography (IEC), isotopic analysis, sample purification, hyphenated technology

## Abstract

稳定同位素分析是分析化学一项颇具前景的分支,通过精确测定物质的稳定同位素比值,可以追溯物质来源并探究其转化过程。高精度稳定同位素分析技术的进步依赖于新一代质谱仪的不断发展。其中,多接收器电感耦合等离子体质谱(MC-ICP-MS)是近年发展迅速的一种同位素组成测定工具。稳定同位素分析对样品基质十分敏感,复杂基质能严重干扰同位素测定的精密度和准确度。这对MC-ICP-MS的样品净化提出了极高要求,目前也是同位素分析领域的热点问题。该文聚焦于近年来MC-ICP-MS在样品净化及仪器联用方法方面的相关研究进展,并展望了MC-ICP-MS稳定同位素分析的应用前景。

## Abstract (en)

Stable isotopic analysis is an important branch of analytical chemistry. Accurate determination of the stable isotopic compositions of substances is critical for tracing their sources and investigating their transformation processes. The new generation of mass spectrometry technology has greatly facilitated the development of high-precision stable isotopic analysis methods. In particular, multi-collector inductively coupled plasma mass spectrometry (MC-ICP-MS) has emerged as a powerful tool for isotopic composition determination. However, isotopic analysis by MC-ICP-MS is highly sensitive to the sample matrix, which may compromise the precision and accuracy of the analysis. Therefore, it is particularly important to reduce the sample matrix effect using efficient sample purification techniques. This article summarizes the recent progress in sample purification and instrument hyphenation methods for MC-ICP-MS, and provides perspectives on the future application of MC-ICP-MS in different fields.

目前,稳定同位素分析已经广泛应用于地球科学、环境科学等领域,为传统分析技术难以解决的问题提供了一种新手段。热电离质谱(thermal-ionization mass spectrometer, TIMS)、二次离子质谱(secondary ion mass spectroscopy, SIMS)以及多接收器电感耦合等离子体质谱(MC-ICP-MS)等新一代质谱技术的发展,为以金属元素为代表的非传统稳定同位素的高精度分析奠定了基础。其中,MC-ICP-MS已成为非传统稳定同位素分析的主流仪器,它在电感耦合等离子体质谱的基础上,增加了法拉第杯离子检测器和双聚焦质量分析器等核心部件,使其在离子化效率和质量分辨率等方面的性能相较于其他同位素质谱得到较大提升。

稳定同位素分析对分析精度的要求极高(一般要求2SD<0.5‰),而高精度同位素分析对样品基质极为敏感,基质效应能够对分析信号灵敏度、准确度和稳定性产生严重干扰。随着非传统稳定同位素技术的发展,MC-ICP-MS应用的环境体系越来越复杂,因此消除同位素分析中的样品基质效应显得尤为重要,这对MC-ICP-MS样品净化技术提出了更高的要求。目前主流的样品净化方法依赖于柱色谱方法。本文聚焦于近年来MC-ICP-MS在样品净化及仪器应用方面的相关研究进展,并展望了MC-ICP-MS的应用前景。

## 1 MC-ICP-MS样品前处理技术

消除样品基质效应主要是指干扰离子(多原子和同质异位素)的去除,这一过程主要通过柱色谱法来实现。选择不同元素富集纯化方法的主要依据是离子交换树脂的材料性质,根据不同元素在不同树脂上吸附力不同,选择合适的酸分步洗脱。尽管柱色谱法具有提纯富集目标元素的能力,但同时存在花费高、操作流程复杂、耗时长、耗酸量高等不足。为了解决这些问题,研究人员不断优化各类元素的纯化富集方法。近年来,MC-ICP-MS样品净化技术取得了快速发展^[1]^。

MC-ICP-MS样品分析通常需要液体进样,且容易受到基质效应的影响。因此,样品前处理一般需要消解和纯化两个步骤来完成样品溶解和基质效应的去除。消解过程的难点在于难溶介质的溶解和较大量危险浓酸的使用。为了克服这些问题,需要开发更加简易高效的消解方法。针对重晶石硫酸钡(BaSO_4_)的难溶性问题,黄方课题组^[2]^发展了一种BaSO_4_简易消解方法(水提法),实现了BaSO_4_中Ba同位素组成的准确测定。重晶石溶解一般采用碳酸钠置换法,用碳酸根( ${\mathrm{CO}}_{3}^{2-}$ )置换硫酸根( ${\mathrm{SO}}_{4}^{2-}$ )后获得易溶于盐酸(HCl)的碳酸钡(BaCO_3_),该方法使用超纯水代替碳酸钠置换Ba元素,在保证Ba同位素测定精度的同时,缩短了化学流程,提高了分析效率。

对于钼(Mo)元素的柱色谱纯化方法,胡兆初课题组^[3]^基于使用辛基苯基-*N*,*N*-二异丁基甲酰甲基氧膦和磷酸三丁酯协同萃取的树脂(TRU树脂)实现了复杂基质中Mo元素的直接分离。传统的Mo元素纯化多使用两柱分离法,其操作烦琐,并且需要复杂的洗脱酸种类和流程才能获得高纯度的Mo溶液,而该方法仅使用单柱进行分离操作,在硝酸(HNO_3_)清洗柱料后仅需HCl洗脱,即可实现Mo元素的纯化及富集,简化了操作流程,并避免了过氧化氢(H_2_O_2_)和氢氟酸(HF)的使用。Mo元素分析对样品量(1~5 g)有一定要求,因此低Mo含量火成岩的高精度Mo同位素分析存在一定挑战性。对此,李杰课题组^[4]^建立了两种Mo元素纯化方法,其中单柱法适用于一般的低Mo含量样品,通过调整*N*-苯甲酰-*N*-苯基羟胺(BPHA)树脂用量,改变洗脱酸的种类和浓度来优化分离效率。对于干扰元素(如铁等)含量过高的低Mo含量样品,则仍需使用双柱法进行分离,不同的是,首先用AG1-X8阴离子交换树脂分离Fe等干扰离子,过程中用2 mol/L HNO_3_洗脱Mo元素,之后将洗脱液与0.2 mol/L HF直接混合即可进行第二步纯化(BPHA树脂),避免了常规方法中的溶液蒸干环节,在缩短实验周期的同时避免了蒸干过程中交叉污染的可能。

对于其他元素,朱建明课题组^[5]^针对含有复杂基质的低镉(Cd)含量样品优化了Cd元素的纯化方法(基于AGMP-1M阴离子交换树脂),降低了流程空白(<0.1 ng Cd)和样品分析用量(单次进样量5 ng Cd),实现了痕量Cd同位素的高精度分析(RSD<0.08‰)。韩贵琳课题组^[6]^改进了纯化钾(K)元素的单柱分离方法,使用更高交联度(12%)和负载量(1.6 meq/mL)的AG50W-X12阳离子交换树脂洗脱富集K元素,从而一定程度上减少了复杂基质对K同位素分析的干扰。

此外,常规元素纯化技术通常依靠重力使溶液流过色谱柱而被收集,这一过程耗时较长。针对这一问题,Mahan等^[7]^提出了一种新的思路(SpinChem^TM^方法),设计了可用于离心的色谱柱,从而增强了离子交换色谱的驱动力,提高了过柱效率,并成功用于锌(Zn)元素的分离纯化和稳定同位素分析。

## 2 MC-ICP-MS仪器性能优化

MC-ICP-MS由进样系统、离子源、质量分析器和离子检测器等多个部分组成。其中,法拉第杯离子放大器是离子检测器部分的关键部件,起到放大电流信号的作用,从而实现对极小离子束的低噪分析。相应地,如果过度提高放大器的放大倍数,在提升仪器分析灵敏度的同时信号噪声也将随之升高,因此如何更好地平衡灵敏度和信噪比是放大器性能优化的一个重要考虑因素。近期,研究人员在离子检测器优化方面取得了一些进展。MC-ICP-MS的放大器常采用10^11^~10^12^ Ω电阻器(电阻值越高,放大倍数越高)。为进一步提升仪器灵敏度,Creech等^[8]^测试了10^13^ Ω电阻器在同位素分析中的信号放大性能。他们在MC-ICP-MS上配备了5个10^13^ Ω电阻器,使用双稀释剂法对铂(Pt)同位素分析进行校正,通过参数优化实现了Pt同位素组成的高灵敏度分析,所需样品量为常规10^13^ Ω电阻器测定的1/50,同时测试结果的不确定度仅增加约3~6倍,较好地平衡了灵敏度和信噪比的问题。这一结果证明了10^13^ Ω电阻器可以应用于稳定同位素的高精度分析。此外,Dellinger等^[9]^也验证了10^13^ Ω电阻器在提高铼(Re)同位素分析灵敏度方面的优异性能。

## 3 MC-ICP-MS与其他进样系统的联用

MC-ICP-MS与各类进样系统的联用一直是稳定同位素分析的前沿研究领域,进一步扩展联用系统的应用领域和克服在线联用系统分析精度和稳定度较低等不足是该领域的两个热点问题。目前,常用的联用进样系统有激光剥蚀(laser ablation, LA)、气相色谱(GC)和液相色谱(LC)进样系统。其中,LA是一种固体取样技术,利用激光脉冲提供高密度能量,可以从固体样品表面剥蚀出细颗粒态样品,并通过N_2_吹扫将细颗粒样品输入质谱仪进行分析。因此,LA-MC-ICP-MS可以避免复杂的固体样品前处理过程,实现对固体样品进行直接(成像)同位素分析。近期,Gonzalez等^[10]^建立了基于纳秒(nanosecond, ns)-LA与MC-ICP-MS联用(ns-LA-MC-ICP-MS)在线分析微米级陨石铁(Fe)同位素组成的方法,通过对瞬态Fe同位素信号进行积分获得准确的Fe同位素比值。这一研究为LA-MC-ICP-MS在微米级物质原位同位素分析领域的应用提供了技术支持。与ns-LA相比,飞秒(femtosecond, fs)-LA具有更短的脉冲信号和更高的瞬时功率,因此可以提高样品剥蚀效率,降低剥蚀颗粒物粒径和剥蚀过程的热效应。胡兆初课题组^[11]^使用fs-LA-MC-ICP-MS精准测定了硅酸岩样品中的硅(Si)同位素组成,利用fs-LA获得了稳定且均质的熔融硅酸岩细颗粒,进一步提高了进样过程的稳定性和分析精度。Desaulty等^[12]^将梯度扩散薄膜技术(diffusive gradients in thin films, DGT)与LA-MC-ICP-MS进行结合,建立了快速测定自然水体中铅(Pb)同位素组成的分析方法。游离Pb离子在梯度扩散薄膜装置中以自由扩散的方式穿过扩散层后被固定膜(Chelex螯合树脂)捕获,然后使用LA-MC-ICP-MS直接剥蚀固定膜可实现Pb同位素组成的原位分析。DGT采样器的使用为LA-MC-ICP-MS分析液态介质中的金属同位素组成提供了方法。

色谱-质谱在线联用可以将色谱优异的分离能力和质谱强大的分析功能结合起来,同时实现复杂基质中目标物的在线分离和定性定量分析。其中,GC-MC-ICP-MS联用系统常用于分析气态物质(如有机物中的气态汞、硫、卤族同位素等)的瞬时信号,从而获得同位素比值。色谱-质谱系统对稳定的信号转化传输有很高的要求,针对此,Thermo Fisher公司开发了一种在线联用技术,通过GCI 300传输线将Trace 1310气相色谱仪与Neptune XT MC-ICP-MS联用,同时实现了有机化合物中低Pb含量的Pb元素形态和同位素分析^[13]^。近期,为了研究铬(Cr)元素在氧化过程中的同位素分馏机理,Karasiński等^[14]^发展了高效液相色谱与MC-ICP-MS的联用体系(HPLC-MC-ICP-MS),实现了对氧化过程中不同形态Cr元素Cr(Ⅲ)和Cr(Ⅵ)的分离及同位素组成的在线追踪,避免了耗时的离线柱分离过程。需要指出的是,色谱-质谱技术的在线同位素分析仍存在一些应用难点,比如瞬时信号稳定性较差和易受样品基质干扰等问题,其分析精度低于常规离线分析,因此仍存在进一步优化的空间。

## 4 MC-ICP-MS的一些新应用

MC-ICP-MS在样品净化及仪器联用方面的进展推动了稳定同位素技术在新的应用领域的拓展。元素的稳定同位素组成能够指示该元素特定的来源或过程信息,表现出一定的指纹特性,因此又被称为“同位素指纹”(isotopic fingerprints)。利用稳定同位素的特征指纹,可以追溯物质的环境迁移转化及归趋规律,并探究相关的机理。本课题组^[15]^利用柱色谱净化和MC-ICP-MS高精度银(Ag)同位素分析技术发现自然水环境体系中的纳米银在自然转化过程中发生了稳定Ag同位素分馏现象,并根据Ag同位素组成的稳定变化揭示了纳米银的迁移转化途径和机理。这一发现表明MC-ICP-MS在环境纳米领域也具有较好的应用前景。此外,基于离子交换柱色谱和MC-ICP-MS的高精度硅(Si)同位素分析,发现不同来源的纳米二氧化硅(SiO_2_)颗粒具有特征的硅-氧(Si-O)双同位素指纹差异,结合机器学习模型,可以实现对天然和人造SiO_2_颗粒的区分,鉴别准确度可达93%^[16]^。利用MC-ICP-MS的高精度Si同位素组成分析还可以追溯大气细颗粒物(PM_2.5_)的来源及成因^[17,18,19]^。

大气细颗粒物污染与多种疾病有关。近期研究发现,在非职业暴露的人体胸腔积液及血液中可以提取到多种超细颗粒物(粒径<0.1 μm),并利用柱色谱净化和MC-ICP-MS高精度Fe同位素分析,结合特征元素指纹和超高分辨结构指纹,证明其主要来自于外源性的大气燃烧源颗粒物^[20]^。此外,利用MC-ICP-MS同位素分析可以追踪血铅暴露的来源^[21]^。使用MC-ICP-MS分析抗生素暴露小鼠肠道中的铜(Cu)同位素组成,发现其分馏发生改变,同时铜转运蛋白表达也发生改变,表明肠道菌群可能能够影响肠道的铜吸收转运^[22]^。这些研究表明了MC-ICP-MS分析在环境及医学领域的应用潜力。

近期有研究发现,史前时期哺乳动物化石的牙釉质Zn和钙(Ca)稳定同位素分馏有效保留了原始的古饮食信息,利用MC-ICP-MS高精度Zn和Ca同位素分析可以评估古代人类和动物群之间的饮食及食物网中的营养关系,进而探究古代人类进化的相关信息^[23,24]^。这表明了MC-ICP-MS在生物进化等研究领域的应用。利用MC-ICP-MS对Fe同位素的高精度分析能力,可以揭示富Fe和溶解性有机质中有机胶体生物降解和光降解过程中的Fe元素的转化行为,这体现了MC-ICP-MS在研究物质迁移转化过程中的应用潜力^[25]^。表1总结了近期一些MC-ICP-MS的典型应用。

**表 1:** MC-ICP-MS同位素分析的近期典型应用

Isotopes	Separation media		Standard material		Sample matrix		Instrumentation		Reference	
^100^Mo,^98^Mo, ^97^Mo,^95^Mo	TRU Spec resin		NIST SRM 3134, BCR-2, BHVO-2, AGV-2, W-2, SGR-1, NOD-P-1, NOD-A-1		rock reference materials, seawater		MC-ICP-MS		[3]	
^100^Mo,^98^Mo, ^97^Mo,^95^Mo	AG1-X8 (100-200 mesh), BPAH resin		NIST SRM 3134, BCR-2		igneous rock		MC-ICP-MS		[4]	
^114^Cd,^113^Cd, ^111^Cd,^110^Cd	AGMP-1M (100-200 mesh)		NIST 3108, BAM-I012, Spex-Cd		rock reference materials		MC-ICP-MS		[5]	
^41^K,^39^K	AG50W-X12 (200-400 mesh)		NIST SRM 3141a, GBW (E) 081590, BCR-2, BHVO-2, AGV-2, JG-2, GSP-2, DNC-1a, RGM-2, SDC-1, W-2a, GSS-2, GSS-3, GSS-4, GSS-6, GSS-8, JMS-2, GSD-6, GSD-11, GSD-12, GSD-14, GSV-2, GSB-6, GSB-14		rock, soil, sediment, plants		MC-ICP-MS		[6]	
^68^Zn,^66^Zn, ^64^Zn	SpinChem^TM^, AG1-X8 (200-400 mesh)		ACR-Zn, IRMM-3702 Zn, BCR-2, BCR-2, BIR-1, BHVO-1, BHVO-2, G-2		geological reference materials, bone ash		MC-ICP-MS		[7]	
^57^Fe,^56^Fe, ^54^Fe		AGMP-1M (100-200 mesh)		KL2-G, ML3B-G, GOR128-G, GOR132-G, ATHO-G, StHs6/80-G, T1-G, BCR-2G, BHVO-2G		micrometeorites		ns-LA-MC-ICP-MS		[10]	
^30^Si,^29^Si, ^28^Si		-		BHVO-2, BCR-2, AGV-2, GSP-2, GSR-1, RGM-2, BHVO-2G, NIST NBS-28		bulk silicate rock		fs-LA-MC-ICP-MS		[11]	
^208^Pb,^207^Pb, ^206^Pb,^204^Pb		-		NIST 610		natural water		DGT/LA-MC-ICP-MS		[12]	
^208^Pb,^207^Pb, ^206^Pb		-		SA1, c3		dust samples		GC-MC-ICP-MS		[13]	
^53^Cr,^50^Cr		-		SRM 979		natural water		LC-MC-ICP-MS		[14]	
^109^Ag,^107^Ag		-		NIST SRM 978a		AgNPs, Ag^+^ ions		MC-ICP-MS		[15]	
^30^Si,^29^Si, ^28^Si,^18^O,^16^O		Dowex 50-X12 (200-400 mesh)		NIST SRM 8546, IRMM 017		natural and anthropogenic SiO_2_ NPs		MC-ICP-MS		[16]	
^30^Si,^29^Si,^28^Si		Dowex 50WX8 (200-400 mesh)		IRMM-017, NIST-1648a, NIST SRM-8546		fine particulate matter		MC-ICP-MS		[17,18]	
^30^Si,^29^Si,^28^Si		Dowex 50-X12 (200-400 mesh)		SRM-8546, IRMM-017		fine particulate matter		MC-ICP-MS		[19]	
^57^Fe,^56^Fe,^54^Fe		AGMP-1M (100-200 mesh)		CAG-Fe		NPs extracted from pleural effusion		MC-ICP-MS		[20]	
^208^Pb,^207^Pb, ^206^Pb,^204^Pb		AG1-X8 (100-200 mesh)		SRM-981, BCR-2		human whole blood samples		MC-ICP-MS		[21]	
^65^Cu,^63^Cu		Cu-specific resin		SRM-986, SRM-976, AE633		mice intestinal tissues		MC-ICP-MS		[22]	
^66^Zn,^64^Zn		AG1-X8 (200-400 mesh)		NIST SRM 1400, AZE bone		fossil teeth		MC-ICP-MS		[23]	
^44^Ca,^43^Ca,^42^Ca		AG50X-W12		NIST SRM 1486, SRM915a		human enamel		MC-ICP-MS		[24]	
^57^Fe,^54^Fe		AG1-X4 (200-400 mesh)		IRMM-014		humic acid containing waters		MC-ICP-MS		[25]	

NPs: nanoparticles; -: not mentioned.

## 5 结论与展望

目前,MC-ICP-MS涉及的样品已经从岩石等地质样品逐渐扩展到成分更加复杂的环境和生物样品,而随着分析样品基质复杂性的提高,对MC-ICP-MS样品净化及仪器分析技术提出了更高的要求。目前,MC-ICP-MS分析技术正朝着流程化、自动化、高效率以及多联用方式的方向发展。未来,如何更加高效地消解和纯化复杂基质样品中的目标元素,尤其是对于高有机质含量的生物样品,如何在同时含有大量有机质及无机干扰元素的样品中进行目标分析元素的分离纯化,如何增强现有柱色谱样品纯化技术的效率和通量,将是拓展MC-ICP-MS应用领域的关键问题。
